# Structural incorporation into goethite fractionates rare earth elements

**DOI:** 10.1039/d5ra04022a

**Published:** 2025-08-14

**Authors:** Sebastian T. Mergelsberg, Alex J. Kugler, Elaine D. Flynn, Eric J. Bylaska, Duo Song, Jeffrey G. Catalano, Eugene S. Ilton

**Affiliations:** a Pacific Northwest National Laboratory Richland Washington 99354 USA sebastian.mergelsberg@pnnl.gov eugene.ilton@pnnl.gov; b Department of Earth, Environmental, and Planetary Sciences, Washington University St. Louis Missouri 63130 USA catalano@wustl.edu

## Abstract

Up to 20% of rare earth elements (REEs) in ion adsorption deposits (IADs) are associated with iron oxide minerals, primarily goethite. Often termed “non-extractable”, goethite-hosted REEs are thought to be structurally incorporated into the mineral lattice. The large mismatch in size and charge density between REEs (ionic radius, *r* = 0.86–1.03 Å) and Fe^3+^ (*r* = 0.65 Å), however, makes direct substitution energetically unfavorable. To determine REE compatibility with and incorporation into goethite on the atomic level, we used X-ray pair distribution function analysis (PDF) and L_III_-edge extended X-ray absorption fine structure (EXAFS) spectroscopy. The compatibility of REEs with goethite and the precursor ferrihydrite (FH) is Lu ≥ Yb ≫ Dy > Nd for both phases. Nd and Dy primarily form secondary amorphous phases, with <30% Nd and Dy incorporated into goethite. In the FH precursor at pH 6.8, Yb and Lu assumed a local REE-OOH like structure with next nearest neighbor Fe. The Yb, Lu, and Nd-FH samples were also matured at ambient conditions for 100 days; despite the presence of only ∼5% goethite, Lu and Yb were 42% and 100% in goethite-like structural environments, respectively, whereas the PDF and EXAFS of Nd showed little evidence of any incorporation. Using *ab initio* molecular dynamics (AIMD) to model the EXAFS, we determined the presence of protonated Fe vacancies, edge-sharing with structural Lu and Yb, likely helped accommodate these REEs in the goethite structure. Incorporation into Fe oxyhydroxides thus potentially fractionates the REEs during weathering associated with formation of lateritic and ion adsorption deposits.

## Abstract 2

This study demonstrates REE fractionation through structural incorporation into iron minerals, like goethite, and the underlying physical mechanism.

## Graphical abstract

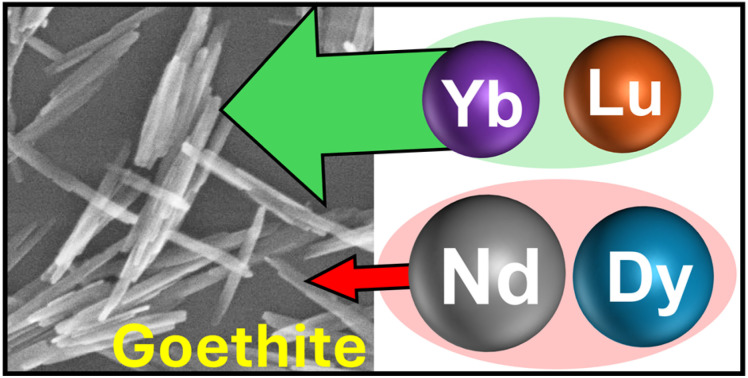

## Introduction

Iron (oxyhydr)oxide minerals host rare earth elements (REEs) in an array of weathering, resource, and sedimentary environments. Regolith-hosted REE deposits often contain a significant fraction (<20%) of these elements in iron oxide phases not extractable *via* cation exchange methods.^1–6^ Formation of these REE-rich iron oxides has been observed directly under variable redox conditions in some regolith deposits.^7^ In some weathering systems, iron oxides serve as dominant controls on REEs, with up to 95% of REEs associated with iron oxides in saprolite profiles formed over gneiss.^8^ This extends to large lateritic weathering deposits, such as bauxites^9^ and the Mount Weld REE deposit,^10^ both of which feature substantial iron oxide-hosted REEs. Beyond weathering deposits, REEs in iron oxide mineral structures have been observed in the hydrothermal Olympic Dam^11^ and some sedimentary iron formation in minor^12^ to major^13^ amounts.

The association of REEs with iron oxides in these more recalcitrant components generally produces fractionations in favor of heavy REEs (HREEs), consistent with the substantial contraction in ionic radius across the lanthanide series.^14^ Regolith-hosted REE deposits show relative HREE enrichment in the iron oxide pool compared to ion exchanged REEs associated with clays,^3–6^ although weak fractionation towards light REEs (LREEs) is reported in some locations.^5^ Iron oxides in bauxites show relative enrichment in HREEs compared to the bulk material or discrete aluminum oxides.^9^ Goethite and hematite from an iron formation shows substantial relative HREE enrichment.^13^ Outliers to these trends are iron oxides in the Mount Weld and Olympic Dam deposits, which strongly concentrate LREEs over HREEs, but this likely caused by nanoscale REE-phosphate inclusions and not fractionation *via* lattice substitution of REEs.^10,11^ In general, however, the extents of fractionation are typically determined using chemical extractions and it is thus unknown whether REEs associated with iron oxides exist in adsorbed, occluded, or incorporated forms.

Limited experimental studies have indicated the structural incorporation of REEs in iron oxides, but observations generally suggest that this mechanism favors HREEs because of their closer match to Fe^3+^ cation radius. Coprecipitation with ferrihydrite (FH) has been interpreted by some to incorporate REEs in the mineral structure^19^ but has also been shown to result in primarily REE adsorption.^15,16,20,21^ The adsorption of REEs to iron oxides weakly fractionates towards HREEs.^15–18^ Coprecipitation of REEs with ferrihydrite and goethite at elevated temperature (75 to 80 °C) show greater retention of HREEs, although structural incorporation was not demonstrated.^22,23^ Conversion of ferrihydrite into various mixtures of more crystalline iron oxides through reductive^24,25^ and thermal pathways^19^ generally observe preferential HREE binding, but evidence for incorporation is indirect, obtained *via* sequential extractions. Prolonged (12 years) aging at RT of Lu(iii)-adsorbed ferrihydrite results in the REE being incorporated into goethite but excluded from hematite.^26,27^ This integrated spectroscopic, microscopy, and computational study provided the most robust evidence to date of the structural incorporation of a REE into an iron oxide structure.^26,27^ Collectively, prior work suggests that ion size differences across the REE series are likely the primary control on variations in REE incorporation into iron oxide structures. This is further supported by the high affinity of the smaller but chemically analogous Sc(iii) for incorporation into iron oxides.^28–30^ Aside from Lu,^26,27^ a critical challenge is the lack of direct evidence for REE substitution into iron oxide minerals, and identification of possible substitution mechanisms, which hinders the ability to assess the importance of this REE retention pathway in weathering, resource, and sedimentary systems.

In this contribution we determined the extent and mechanisms of Neodymium (Nd), Dysprosium (Dy), Ytterbium (Yb), and Lutetium (Lu) incorporation into FH and goethite *via* two formation pathways: (1) coprecipitation of FH and REEs by hydrolysis of Fe(iii) to pH 11 followed by maturation at 60 °C; (2) initial coprecipitation of REEs and FH at circumneutral pH (pH 6.8) followed by 100 days of aging at room temperature. The former establishes a baseline for pure REE-goethite samples but is an unlikely incorporation pathway in most environments because of the temperature and pH involved. The latter follows a more realistic incorporation pathway,^26,27^ akin to the approach taken by Finck *et al.*,^27^ but involving initial REE-FH coprecipitation instead of initial adsorption of the REEs to FH. The extent of incorporation in goethite and the structural mechanism were quantified using sequential dissolution, extended X-ray absorption fine structure (EXAFS) spectroscopy (Nd, Yb and Lu), high-energy X-ray diffraction (heXRD), STEM-HAADF (Yb-goethite), and X-ray PDF analysis. *Ab initio* molecular dynamics (AIMD) was applied to create synthetic EXAFS spectra for different substitution configurations (structural position and associated defects) which were used for linear combination fitting (lcf) of the experimental EXAFS spectra, an approach we refer to as AIMD-informed EXAFS. The advantage of this approach compared to the traditional use of shell-by-shell fitting is that the configuration and thermal disorder are decoupled, which avoids correlation of the coordination number (CN) and the thermal disorder (*σ*^2^) fitting parameters. This methodology has enabled prior work to determine that semi-compatible impurities often occur in multiple configurations in a single mineral, an observation not possible with traditional EXAFS data analysis approaches.^31–39^

## Methods and materials

### Sample synthesis

For all undoped samples, 62.5 mmol of iron nitrate nonahydrate were dissolved in 450 mL de-gassed 18.2 MΩ cm ultrapure water. For doped materials, 61.25 mmol of iron and 1.25 mmol of REEs were added as the nitrate salts, producing a target solution with REE/(Fe + REE) = 2 mol%. The resulting solutions were split into thirds to produce (1) pure ferrihydrite, (2) aged ferrihydrite, (3) pure goethite:

(1). Ferrihydrite (FH). Solutions were titrated to pH 6.80 ± 0.02 using 10 M and 1 M NaOH solutions. Once the solutions reached the final pH, they were washed with de-gassed 18.2 MΩ cm ultrapure water (7×).

(2) Aged ferrihydrite. Similar to pure ferrihydrite, solutions were first titrated to pH 6.80 ± 0.02 using 10 M and 1 M NaOH solutions. The titrated solutions were then aged at room temperature for 100 days, before washing the samples 7× with 18.2 MΩ cm ultrapure water.

(3) Goethite (Goe). The initial metal nitrate solutions were rapidly hydrolyzed under stirring using 250 mmol NaOH over 10 minutes, for a final Fe : Na ratio of 1 : 4. The resulting suspensions were vortexed to ensure even mixing. Samples were aged in a forced convection oven at 60 °C for 3 days followed by centrifugation at 8000*g*, then 3 water washes, one acid wash (pH = 3, HCl), and another 3 water washes. For all washes we used de-gassed 18.2 MΩ cm ultrapure water. Samples were dried in a forced convection oven at 70 °C for 8–14 hours.

Samples from the first and third group are referred to as FH and Goe, respectively, as defined in Table S1. Undoped reference material is identified as RFH and RGoe and the prefixes (Nd, Dy, Yb, and Lu) denote doped materials.

### Mineral compositions

The compositions of REE-incorporated goethite were determined by total digestion of 10 mg of each sample in 15 mL of 20% nitric acid and 5% hydrochloric acid at 70 °C. After the solution cooled to ambient temperature, it was filtered using 0.22 μm MCE filters and diluted with ultrapure water to a nitric acid concentration of 2%. Each digestion was performed in triplicate. Fractional dissolution of each sample was determined by suspending 10 mg of the solid in 100 mL of 4 M hydrochloric acid at 70 °C. Samples were periodically collected until the solids were fully dissolved. The solution aliquots were filtered, diluted, and acidified to 2% nitric acid. Lu, Yb, Dy, Nd, and Fe concentrations were determined using a Thermo Scientific iCap 7400 Duo inductively-coupled plasma optical emission spectrometer.

### Scanning electron microscopy (SEM)

Following synthesis, <1 mg of sample was suspended in 2 mL of ethanol. Ethanol suspensions were vigorously vortexed, a 20 μL sample was deposited on a clean silicon wafer, and the suspension was left to dry for ∼1 hour. All prepared silicon wafers were mounted to aluminum studs using double-sided conductive copper tape, and samples were carbon-coated to ∼7 nm. Samples were imaged to assess particle size and morphology at working distances of ∼4 mm and accelerating voltages of 5–15 kV, using an FEI Helios 600i NanoLab SEM equipped with two secondary electron (SE) detectors: an Everhart–Thornley detector (ETD) and a through-lens-detector (TLD).

### Scanning transmission electron microscopy

To determine the Yb distribution on YbGoe on the atomic scale, microscopy observations were performed with an aberration corrected Thermo-Fisher Themis Z scanning/transmission electron microscope (S/TEM). The observations were performed in scanning mode using a high-angle annular dark-field (HAADF) detector. The microscope was set up with a probe convergence angle of 25 mrad, and the inner detection angle for the HAADF detector of 52 mrad. The acquisition and basic image processing were performed with Thermo-Fisher's Velox software.

### X-ray total scattering measurements

X-ray total scattering data for PDF analysis was acquired at beamline 11-ID-B of the APS using dry powders loaded into 1.1 mm ID polyimide capillaries. X-ray scattering (86.5 keV; *λ* = 0.1432 Å) was measured at a sample-to-detector distance of 245 mm, which was calibrated using a diluted CeO_2_ standard (ZNST 674b). Reciprocal-space data were integrated from 2D to 1D using the GSAS-II software and 1300 bins,^40^ with limited polygon and threshold masks.^41^ PDF profiles were calculated from the data in PDFgetX3 (ref. 42) using *q*_min_ = 0.7 Å, *q*_max_ = 22.4 Å, and *q*_maxinst_ = 29.4 Å. High-energy XRD (heXRD) was acquired at sample-to-detector distances of 1000 and 1200 mm. GSAS-II was also used to refine the goethite lattice parameters for each heXRD sample.

### X-ray absorption spectroscopy

X-ray absorption spectroscopy (XAS) measurements at the Nd, Yb, and Lu L_3_-edges were performed at the Stanford Synchrotron Radiation Lightsource (SSRL) and the National Synchrotron Light Source II (NSLS-II). EXAFS was not measured for DyFH and DyGoe because of high background signals from Fe fluorescence. Samples were packed into Delrin holders for measurements at SSRL and aluminum holders for NSLS-II; both were sealed with Kapton tape. Details of both beam line configurations are given in SI Note S1. Fluorescence-yield data were collected using a Vortex 4-element silicon drift detector. Multiple scans of each sample were averaged to improve the signal to noise ratio. Data were averaged and normalized in the SixPack^43^ and Athena^44^ interfaces to IFEFFIT,^45^ followed by extraction of the EXAFS spectrum for analysis.

### Theoretical simulations of EXAFS spectra

All pseudopotential plane-wave (PSPW) DFT simulations were performed with the plane-wave NWPW module contained in the NWChem software package^46–52^ using the DFT PBE96 exchange correlation functional.^53^ AIMD simulations were performed for at least 20 ps for individual YbGoe and LuGoe configurations (AIMD-informed EXAFS was not performed for NdGoe because this sample showed unidentified secondary phases). The system was propagated in time using the Car–Parrinello molecular dynamics (CPMD) scheme.^54,55^

For both the Lu and Yb atoms, projector augmented-wave (PAW) potentials were specifically employed to accurately model the complex electronic structure inherent in these heavier rare earth elements.^37^ The PAW approach is essential for correctly representing the valence electrons and the critical 4f-electron states in Lu and Yb, which standard pseudopotentials often fail to describe accurately. By using PAW potentials with a high cutoff energy of 120 Ry, we ensure precise handling of the 4f states that are important in these heavy elements. This level of precision is crucial for generating reliable and accurate EXAFS spectra and for making detailed structural interpretations of the local environments of these REEs. The PAW method enables a more comprehensive description of the electronic environment around Lu and Yb, ultimately yielding a better match to experimental data.

The host structure was a 96-atom orthorhombic FeOOH goethite super-cell (*a* = 10.0667 Å, *b* = 9.1552 Å; *c* = 9.2041 Å; *α* = *β* = *γ* = 90°) obtained from optimizing the unit cell (1 × 3 × 2 conventional cell). A spin-penalty scheme was used to initialize the antiferromagnetic configurations, more information is provided in the SI (Note S2).

To generate the L_3_ edge EXAFS spectra for these simulations, snapshots were collected every 30 fs resulting in a pool of ∼620 snapshots for each simulation. An EXAFS spectrum for each of these was calculated independently by FEFF9,^56^ and then averaged for each simulation. The averaging of the computational spectra and lcf to the experimental spectra was done in *k*-space. Our approach closely follows our prior work on cations in aqueous solutions and impurities in the solid state.^31–37,39,57^

## Results and discussion

### REEs in goethite synthesized at 60 °C

#### Bulk properties of synthesis products

Goethite prepared *via* an alkaline synthesis at elevated temperature produced acicular needles and platelets (Fig. S1). The addition of REEs, especially Yb and Lu, resulted in a decreased aspect ratio. Rietveld refinements of the heXRD patterns are consistent with 100% goethite for all samples, with no evidence for secondary crystalline phases (Fig. S2). Fractional dissolution of the acid-washed synthetic REE-goethites shows near-stoichiometric release of Yb and Lu after an initial ∼10% of the total Fe enters solution (Fig. S3). The excess Yb and Lu entering solution during the early stage of dissolution likely derive from the surface (<1 nm deep). In contrast, both Nd and Dy goethite samples display substantial non-stoichiometric release consistent with a separate surface-associated phase (Fig. S4). Further, the interior of goethite particles that dissolve later during fractional dissolution contain low Nd and Dy concentrations (0.2 to 0.5 mol%), in contrast to the greater levels displayed by Yb and Lu (Fig. S4, inset). The dissolution data indicate the order of compatibility of the REEs in goethite is Lu ∼ Yb > Dy > Nd.

**Fig. 1:**
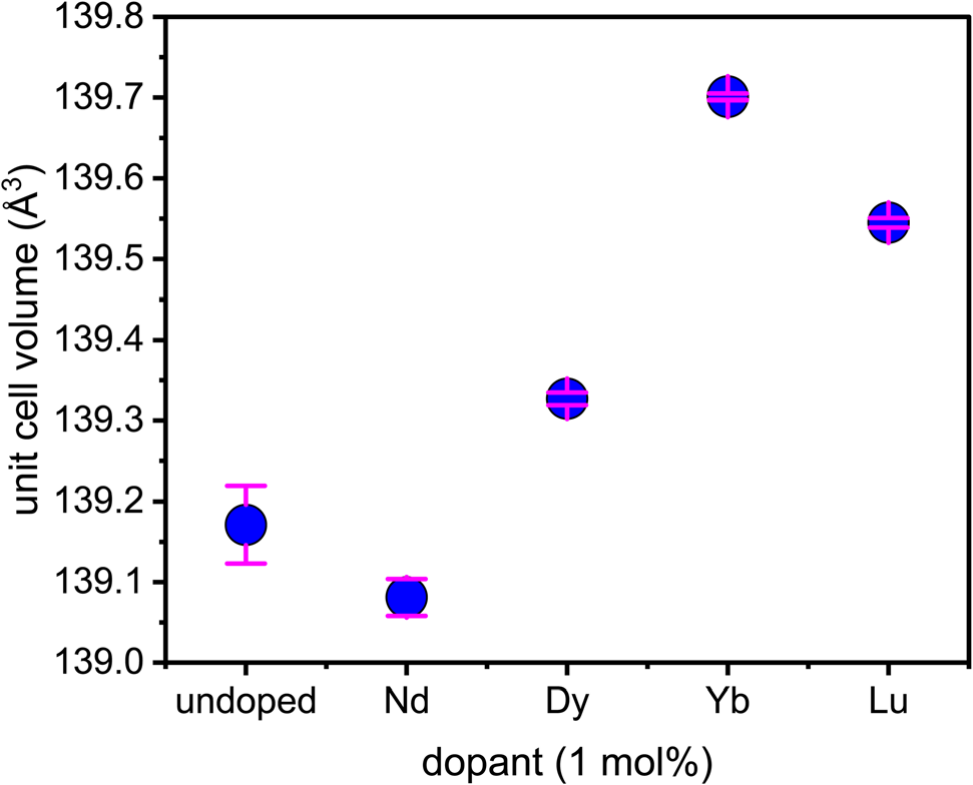
The refined unit cell volume of undoped and REE-doped goethite, as measured by heXRD. Detailed fits and fitting parameters are summarized in Fig. S2 and Table S2. The pink error bars represent the model error. Note that the dopant concentration (1 mole%) is a target value; actual incorporation levels are discussed in the text.

The heXRD data also shows shifts in lattice parameters expected for incorporation of a larger ion (*i.e.*, a REE) into goethite for Dy, Yb and Lu but not Nd (Fig. 1 and Table S2). This suggests that the extent of incorporation into the lattice varied between the light, middle, and heavy REEs, consistent with the dissolution data. In addition, less Dy incorporation compared to Yb and Lu is implied by the smaller increase in the unit cell volume for Dy-goethite despite Dy having an appreciably larger effective ionic radius than the two HREEs.^58^

#### Atom-scale structural effects of REE coprecipitation

**Fig. 2:**
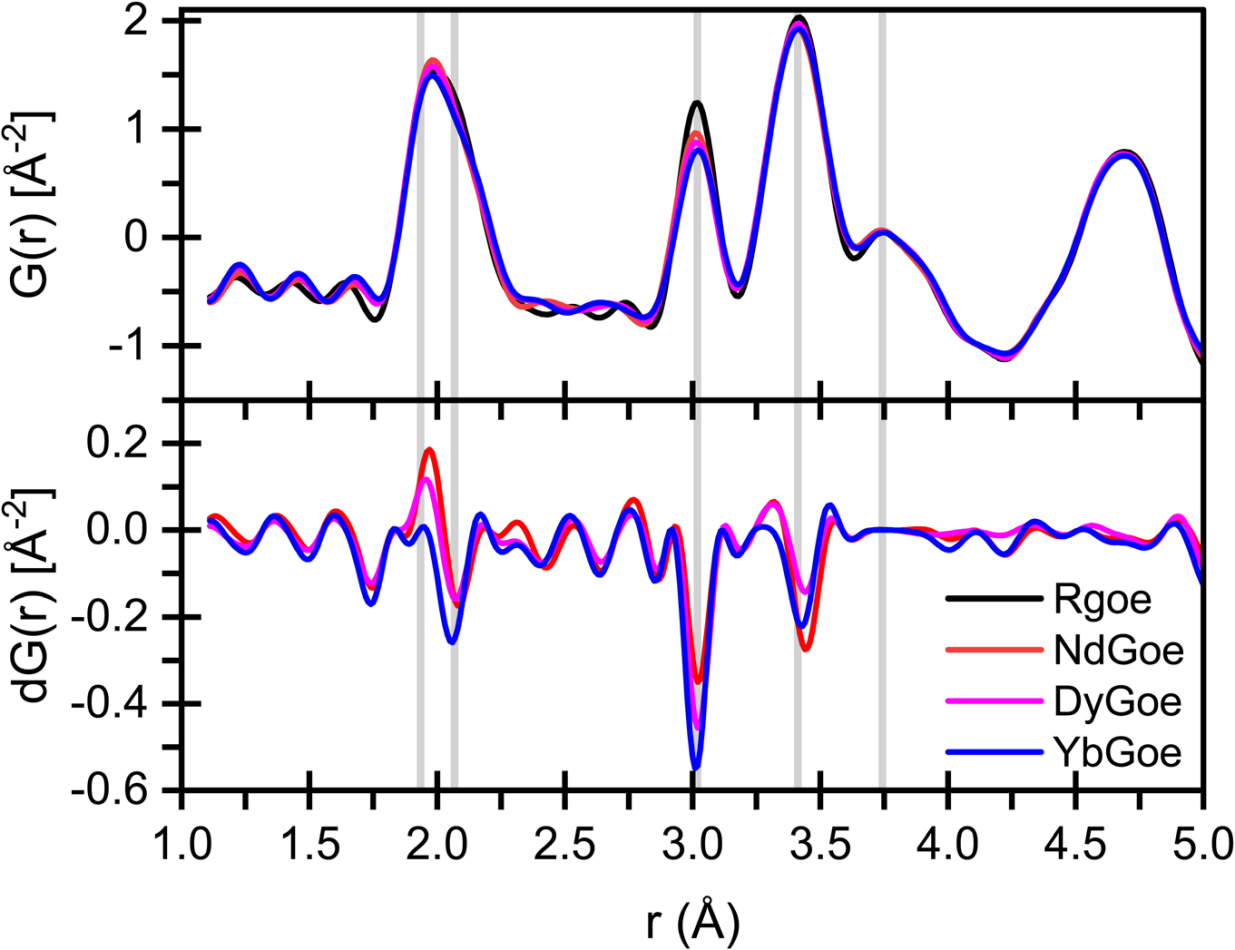
X-ray PDF analysis of the samples. LuGoe data could not be used because two phases were present, the sample and an in-line calibration standard (Fig. S2, peaks in fit residual). The grey lines at 1.936 and 2.068 Å indicate Fe–O and Fe–OH bonds of goethite, respectively, and gray bars at 3.019 and 3.412 Å correspond to Fe–Fe distances. The peak at 3.743 Å is a second coordination shell Fe–O distance. Differential patterns, [d*G*(*r*)], are calculated in real space by subtracting the undoped goethite *G*(*r*) from the doped goethite samples. The analytical error is within the width of the traces.

The PDF analysis of the total X-ray scattering data reveals variations in short-range order associated with the addition of REEs to goethite (Fig. 2). The dPDFs for Nd and Dy show clear evidence for a secondary phase whereas Yb appears mostly incorporated into goethite (Fig. 2). The secondary Nd and Dy phases are likely amorphous as they only show structure in the dPDF up to about 4.5 Å. Amorphous phases would explain the lack of additional peaks in the heXRD patterns (Fig. S2) as well as the excess, non-stoichiometric release of Nd and Dy during dissolution (Fig. S4). The EXAFS spectrum of Nd-goethite shows weak second-shell features (Fig. S5), consistent with limited structural substitution but also precluding identification of the predominant host phase. Nonetheless, the dPDFs for Nd- and Dy-goethite also reveal Fe vacancies independent of the secondary REE phases, which is consistent with Nd and Dy affecting the bulk structure of goethite. The dissolution data suggests that 10% of Nd and 30% Dy are structurally incorporated. This raises the possibility that the observed Fe vacancies may be associated with incorporated Nd and Dy. For Dy, the corresponding lattice expansion of goethite further supports its incorporation. The addition of Nd causes a small decrease of the *a* axis, consistent with prior work,^59^ despite the large ionic radius of this REE. Such a lattice contraction may reflect offsetting effects of a small substitution level of Nd and the introduction of Fe vacancies. In contrast to Nd and Dy, the Yb- and Lu-goethites show no evidence for secondary phases in heXRD. The non-stoichiometry observed for these samples during the first 10% of goethite dissolution thus indicates a near-surface enrichment or incongruent dissolution of the HREEs relative to Fe. The dPDF analysis for Yb-goethite demonstrates substantial Fe vacancies, indicating that these are coupled with its incorporation. To further resolve these differences, we turn to AIMD-informed EXAFS, which uses explicit structural models to determine the structural incorporation of impurities in host minerals.

#### Yb and Lu substitution mechanisms: AIMD-informed EXAFS

**Fig. 3:**
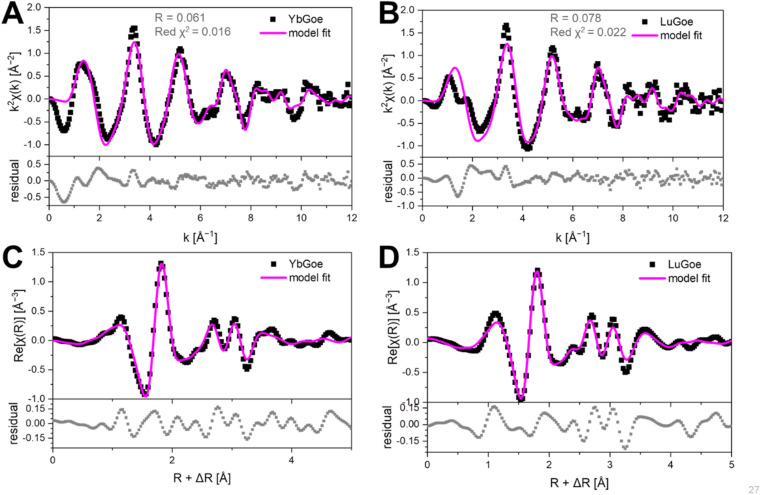
Comparison of AIMD models to experimental EXAFS spectra. (A and C) Linear combination fit of three YbGoe configurations in reciprocal space (A) and the resulting agreement in real space (B). (B and D) Linear combination fit of three LuGoe configurations in reciprocal space (C) and in real space (D). Data in black, model configurations in magenta, and the residual in grey.

The EXAFS spectra of Yb- and Lu-goethite display substantial second-shell features indicative of structural incorporation rather than surface species (Fig. 3). The vast possible configuration space poses a challenge to applying an AIMD-informed EXAFS approach for assessing incorporation mechanisms. Prior work on other incorporated elements, the need to maintain charge balance, and constraints from the PDF analyses enable down-selection to a subset of possible substitution mechanisms to explore. Notably, the dPDF results (Fig. 2) provide clear evidence for Fe vacancies in edge-sharing octahedral sites. Finding configurations for Yb and Lu was further complicated by the appreciable sensitivity of the AIMD trajectories to the magnetic structure of the substituted goethite (see Note S2). In this regard, we explored many different multiplicities, denoted by “mult *n*” in the reported configuration names, which signifies *n* − 1 unpaired electrons. The other numbers in the structure names refer to atom positions for the impurity (*e.g.*, Yb) or vacancies (V). For example, Yb82_84_3c denotes Yb atoms in locations 82 and 84 with 2 unpaired electrons, using permutation “*c*”.

**Fig. 4:**
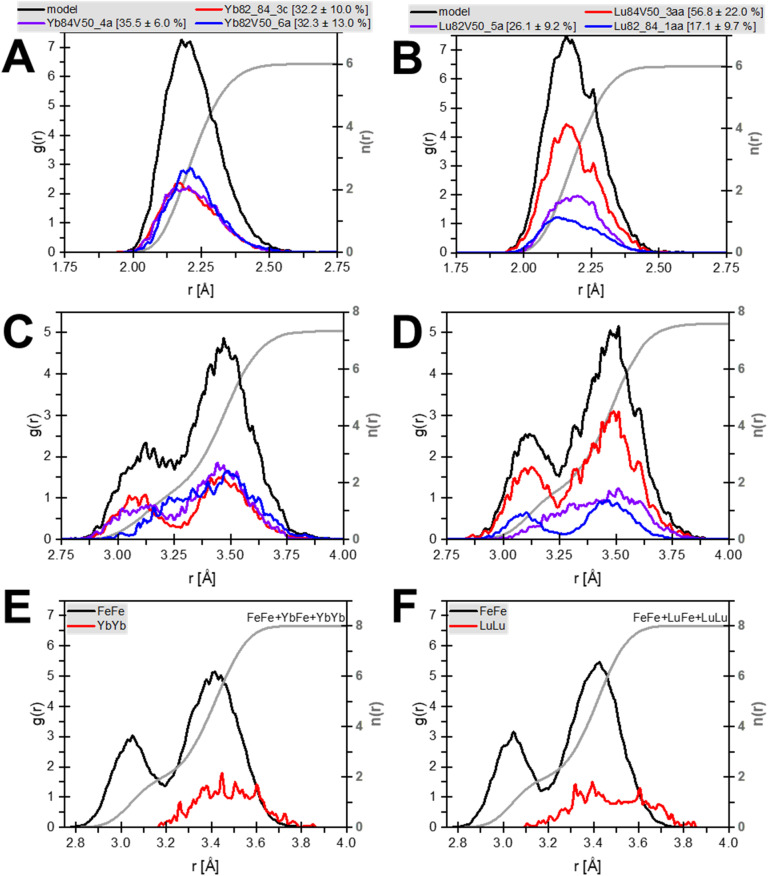
Distribution of distances in AIMD REE-goethite models. (A and B) REE-O distances from all three configurations that carry weight in the fit. The grey line gives the coordination number. (C and D) Distribution of REE-Fe distances. The grey line gives the coordination number of REE-Fe, not including REE–REE pairs. (E and F) Distribution of REE–REE (red) and Fe–Fe (black) for the Yb82_84_3c (C) and Lu82_84_1aa (D) configurations. The grey line gives a combined coordination number of the metal site.

The results confirm that Yb and Lu substitute for Fe in octahedral coordination (Fig. 3 and Table 1), consistent with prior work on Lu-goethite.^26,27^ The average first shell atomic distances (Fig. 4) are 2.23 and 2.20 Å for Yb and Lu, respectively, which are well within the expected range for Yb and Lu in six-fold coordination with oxygen^60–62^ and exactly matches the Lu–O distance previously reported for Lu in goethite.^26,27^ Both Yb- and Lu-goethite display considerable configurational disorder, requiring three different structural motifs per HREE (Table 1). The Yb and Lu configurations are similar but occur in different proportions. This AIMD-informed EXAFS approach thus provides additional insight into the structural complexity underlying REE incorporation than possible using traditional shell-by-shell fitting, which in prior work on Lu-goethite identified a single configuration.^26,27^ Both HREEs occur in two configurations with next nearest neighbor edge-sharing fully protonated octahedral site vacancies (*i.e.*, Fe^3+^ replaced by 3H^+^), which is fully consistent with the dPDF results (Fig. 2). This confluence of AIMD-informed EXAFS and dPDF increases the confidence that configurations with adjacent Fe vacancies occur in Yb- and Lu-goethite. The fit to Yb-goethite also gives substantial weight to a configuration with Yb–O–Yb edge-sharing (Yb82_84), whereas the fit to Lu-goethite yields a relatively minor weight to a Lu–O–Lu edge sharing configuration (Lu82_84).

**Table 1:** The 3-component LCF results for the fits shown in Fig. 3. Fit percentages are tabulated as model % and as the corresponding phase %, accounting for two absorber atoms present in configurations Yb82_84_3c and Lu82_84_1aa

YbGoe model	Model %	Phase %	LuGoe model	Model %	Phase %
Yb82_84_3c	32.2 ± 10.0	19.2 ± 6.0	Lu82_84_1aa	17.1 ± 9.7	9.4 ± 5.3
Yb84V50_4a	35.5 ± 6.0	42.3 ± 7.2	Lu84V50_5a	56.8 ± 22.0	62.1 ± 24.0
Yb82V50_6a	32.3 ± 13.0	38.5 ± 15.5	Lu82V50_3aa	26.1 ± 9.2	28.5 ± 10.1

**Fig. 5:**
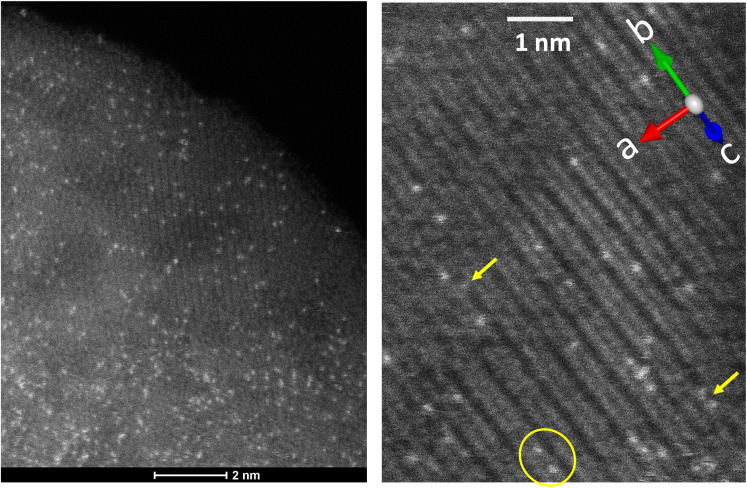
HAADF-STEM images of YbGoe sample. Bright spots indicate the more electron-dense Yb atoms. The diagonal grey lines correspond to the lattice of goethite, with the crystallographic direction indicated by the compass. Likely acid-rinse resistant adsorbed Yb atoms (arrowed) and possible edge-sharing Yb atoms (circled) have been highlighted.

The structural substitution of REE impurities into octahedral sites in goethite is supported by STEM-HAADF images (Fig. 5) that show individual Yb atoms distributed throughout goethite aligned with the octahedral chains (Fig. 5a). No precipitates are observed but Yb is not uniformly distributed, manifesting as clumping at lower resolution (Fig. 5a). At higher spatial resolution (Fig. 5b), the Yb atoms are mostly associated with Fe columns, consistent with substitution of Yb for Fe. No accumulation of Yb atoms was observed near the darker areas, which likely are voids within the goethite structure.^63,64^ Some pairs of Yb atoms are observed separated by ∼2.6–3.7 Å. The two-dimensional view provided by such imaging lacks depth sensitivity, and some visually adjacent Yb atoms may actually be separated by substantial distances. However, the observed Yb–Yb separations are consistent with the edge-sharing configuration identified by AIMD-informed EXAFS. A minority of Yb atoms are associated with low contrast zones (Fig. 5b) that represent oxygens linking the edge-sharing Fe octahedra. These may result from an adsorbed Yb species that resisted desorption in the pH 3 treatment following synthesis. Such surface species may be the source of the initial rapid release of ∼10% of Yb during fractional dissolution (Fig. S1). The curvature of the Yb and Lu dissolution data also indicates some enrichment of Yb and Lu in the near-surface region of the goethite structure. While the STEM-HAADF images do not support substantial enrichment at the particle edge (Fig. 5), Yb concentration in the interior of each goethite crystallite may be lower due to limited depth resolution.

### REEs coprecipitated and aged with ferrihydrite

#### Initial precipitates

**Fig. 6:**
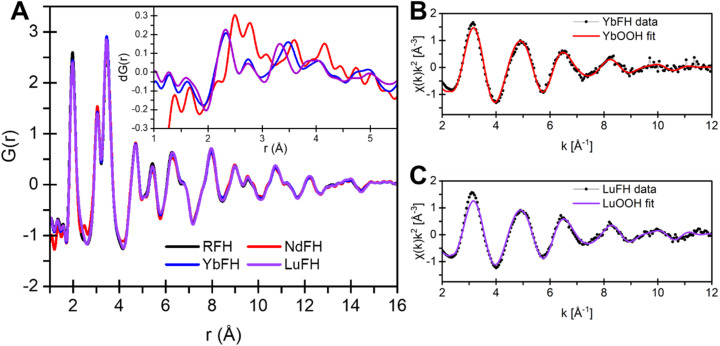
Structural analysis of REE-FH samples. (A) X-ray PDF and differential PDF (inset) of undoped RFH, NdFH, YbFH, and LuFH. DyFH was not measured. (B) Shell-by-shell fit of YbOOH to the YbFH EXAFS data. (C) Shell-by-shell fit of LuOOH to the LuFH EXAFS data. Fit results are reported in Table S3.

Because Goe typically forms *via* FH intermediates, we also coprecipitated Fe with Nd, Yb, or Lu, and without an REE present, to yield only 2-line ferrihydrite (FH) as a detectable product (Fig. S6). Results are well-aligned with 2-line FH reported previously and do not show any evidence for goethite peaks at *q* = 1.4 and 2.6 Å^−1^.^65^ Atom-scale characterization of these materials shows that the PDF for all REE-bearing and REE-free FH precipitates supports formation of only a single 2-line FH phase for YbFH and LuFH samples (Fig. 6a). The differential PDFs, d*G*(*r*), calculated between undoped and Yb- or Lu-bearing FH samples show short range Yb/Lu–O and Yb/Lu–Fe peaks (Fig. 6a, inset). However, both Yb–O and Lu–O distances are around 2.31 Å, indicating 7-fold average coordination.^58,66,67^ The coherence of the Yb and Lu components in the dPDF is only 6 Å, which could indicate that these REEs occur as an amorphous precipitate or subnanometer domains either separate from or within the FH structure. A shell-by-shell fit to the EXAFS data assuming a YbOOH/LuOOH-like local structure (Fig. S7 and Table S3)^66,67^ reproduced the spectra after replacing next nearest neighbor REE with Fe (Fig. 6B and C). The lack of features beyond the first two shells (*i.e.*, limited coherence) and need to replace second-shell REE with Fe in the fitting model (Table S3) indicates that the REE-OOH are unlikely to be separate amorphous phases and are instead associated with FH. In contrast with Yb and Lu, the differential PDF for Nd shows evidence of a short-range ordered Nd precipitate that is not NdOOH (Fig. S8). These data instead indicate that a different, unidentified secondary phase formed and that Nd does not appreciably incorporate into the ferrihydrite structure, under present conditions, as was previously suggested.^59^

#### REE fate during FH aging

**Fig. 7:**
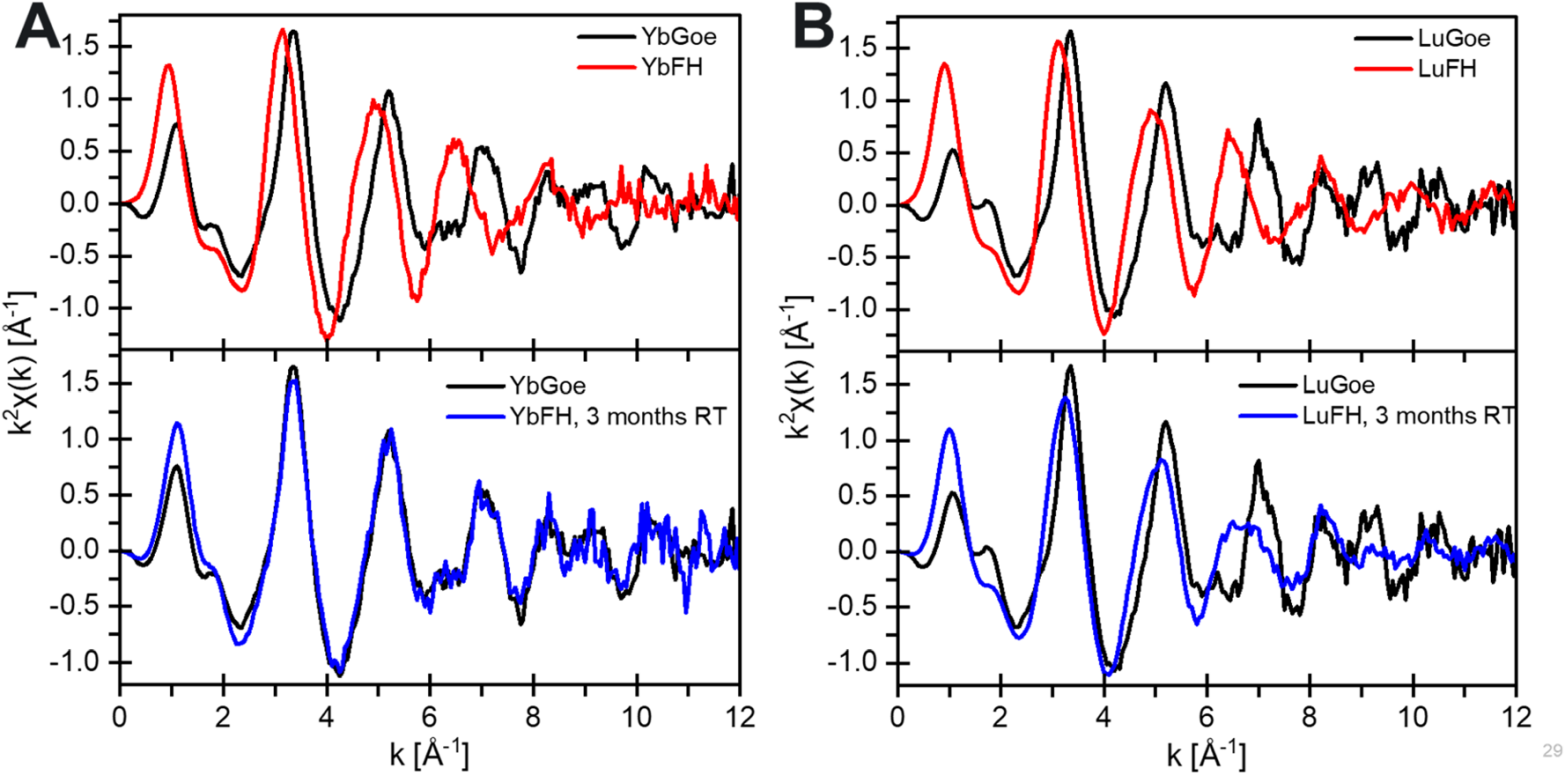
The REE L_III_-edge EXAFS of Yb (A) and Lu (B) samples. The precursor FH in red, the hydrothermal goethite in black, and the RT-aged ferrihydrite in blue.

The Yb-FH, Lu-FH, and Nd-FH coprecipitated samples were aged at ambient temperature over the course of 100 days to assess the impact of partial transformation to goethite on REE fate. Aging resulted in ∼5% conversion of FH to goethite (Fig. S9). However, the EXAFS spectrum of Yb substantially differs from the initial FH precipitate and appears nearly identical to Yb associated with goethite formed at pH 11 and 60 °C (Fig. 7a). This indicates that Yb rapidly takes on a goethite-like local coordination environment during the initial stage of transformation. Further, these results indicate strong partitioning of Yb into goethite over FH. In contrast, the EXAFS spectrum for aged Lu-FH (Fig. 7B) consists of a mixture of 58 ± 1% Lu-FH and 42 ± 1% Lu-goethite (*R* = 0.019, *χ*_ν_^2^ = 0.004). This indicates that Lu adopts a goethite-like coordination environment more slowly than Yb despite having a slightly smaller ionic radius. These observations demonstrate that two neighboring HREEs of similar size and properties uniquely dictate the evolution of their coordination environments when occurring as impurities in iron oxide phases. Unlike the heavy REEs, the EXAFS spectrum of Nd did not evolve substantially after aging (Fig. S10). The first shell feature did not shift in position but weakened in intensity, indicating an increase in disorder without a change in average Nd–O distance. These observations are consistent with the limited incorporation of Nd into goethite during thermal conversion under alkaline conditions.

### Mechanisms controlling REE partitioning into goethite

These results provide new mechanistic insight into the mechanisms through which large REE^3+^ cations are accommodated in the goethite structure. While the HREEs Yb and Lu substitute at 1.67 and 1.21 mol%, respectively, the amount of Dy (MREE) and Nd (LREE) substitution is uncertain because of secondary REE phase formation but significantly lower. Dissolution data suggest that <0.51 mol% Dy and <0.26 mol% Nd enter the goethite structure (Fig. S4), following a relationship with ionic radius of the REE similar to that observed in a prior macroscopic coprecipitation study.^23^ The AIMD-EXAFS approach employed in this study demonstrates the close association of substituted Yb and Lu with protonated iron vacancies. Iron vacancy formation also occurs for goethite co-precipitated with Nd and Dy, as demonstrated by PDF analysis of total X-ray scattering data (Fig. 2). While the local coordination environment of these REEs could not be probed because of secondary phase formation and spectral interference from Fe, we hypothesize that vacancies in the Nd- and Dy-goethite samples are also protonated and adjacent to substituting REEs. This is supported by the consistent association of substituting elements with protonated Fe vacancies in both goethite and hematite.^31,34,68–70^ Validation of the link between Nd/Dy substitution and iron vacancy formation will require synthesis of goethites free of secondary REE phases, likely necessitating a substantially lower REE content to enable complete substitution. This will pose technical challenges to obtaining EXAFS spectra because of the lower REE concentration. Interference from Fe K_α_ and K_β_ fluorescence during fluorescence-yield EXAFS measurements of the MREEs, as occurred for the Dy-goethite sample in this study, will be exacerbated at lower substitution levels and may require X-ray fluorescence detector innovations to obtain data with adequate signal-to-noise ratios.

The favorability of HREE substitution into goethite extends to the slow formation of this iron oxide mineral from a ferrihydrite precursor during aging at ambient temperature for 100 days. While Yb and Lu take on a similar REEOOH-like local structure upon initial coprecipitation with ferrihydrite, they transition to a goethite-like local environment during transformation. For Yb, this transformation is essentially complete after only ∼5% conversion of ferrihydrite to goethite, yielding a near-identical EXAFS spectrum as from Yb-goethite synthesized under alkaline conditions at 60 °C. The transformation of Lu was only partial but the fraction of this REE that enters a goethite-like local structure still substantially exceeded the goethite fraction. The slower kinetics of Lu incorporation into goethite compared to Yb despite the shared local coordination environments lacks a simple mechanistic explanation given the similar ionic radii of the two elements. Substantial differences in electronic structure may favors transformation from the YbOOH-like precursor into goethite compared to the LuOOH-like equivalent. As a closed-shell ion, Lu is likely unaffected by the different magnetic structures inherent to the ferrihydrite and goethite materials. Such electronic and magnetic structure effects are a potentially unique feature of REE interactions with iron oxyhydroxides that cannot occur with aluminum-rich weathering products like gibbsite and clay minerals.

Prior work shows that Lu initially adsorbed to ferrihydrite similarly enters the goethite structure during aging at ambient temperature,^26,27^ although differences in aging time (100 days *versus* 12 years) do not allow comparison of partitioning rates. Unlike Yb and Lu, limited changes in Nd coordination occur during aging of a ferrihydrite coprecipitate in this study (Fig. S5), indicating that negligible Nd incorporation occurred despite a similar extent of goethite formation as for the HREE coprecipitates (∼5 wt%). These new and previous observations suggest that HREEs favor the goethite structure compared to ferrihydrite. When combined with the reported preferential removal of HREEs compared to LREEs during ferrihydrite precipitation,^71^ observations in this study indicate that partitioning into goethite strongly selects for the HREEs. Our findings are also in qualitative agreement with trends in REE structural incorporation inferred during the Fe(ii)-catalyzed transformation of ferrihydrite to more crystalline iron oxides.^24,25^ However, evidence for incorporation in these prior studies rests solely on dilute acid extractions, which our work shows may leave behind a residual adsorbed REE pool. Further, ferrihydrite transformation extents and products varied among the REEs investigated in these prior studies, hindering quantitative assessment of trends in partitioning behavior.

Variations in incorporation trends across the rare earth series during initial ferrihydrite coprecipitation suggests that LREEs and HREEs follow different pathways when associating with iron oxides. A qualitative indicator is the slight decrease in goethite lattice parameters with limited Nd incorporation, consistent with observations by a previous study (Fig. S11).^59^ The distinct initial coordination environment of Nd compared to Yb and Lu during coprecipitation with ferrihydrite (Fig. S8), with a substantially larger and more distorted first coordination shell, may create a barrier to eventual Nd incorporation into goethite by hindering nucleation of Nd-bearing local domains. This inhibitory mechanism induced by distinct initial coordination of Nd *versus* HREEs may also apply to the more rapid goethite synthesis conducted at elevated temperature, which also involves an initial ferrihydrite coprecipitate.

### Implications for REE resources and environmental fate

The controls on REE incorporation into goethite identified in this study, including the role of an REE oxyhydroxide-like precursor state, demonstrate a potential mechanism underlying the fractionation towards HREEs observed for iron oxides in the environment. Regolith-hosted REE deposits and bauxites often contain an iron oxide pool enriched in HREEs.^3–6,9^ Outliers to this trend that show LREE enrichment have been attributed to nanoscale REE-rich inclusions.^10,11^ However, our work shows that amorphous REE phases may also serve as partial hosts for LREEs and MREEs. Acid dissolution displayed faster rates of release for LREE- and MREE-amorphous components compared to the minor fractions structurally-bound in goethite. This suggests that more targeted dissolution approaches may enable LREE/MREE *versus* HREE separations during recovery from iron oxide pools.

Our studies also suggest that coprecipitation with Fe(iii) may be a viable mechanism through which LREEs, MREEs, and HREEs fractionate in the critical zone. Partitioning relationships need to be established to provide a more quantitative prediction of relative fractionation effects, including studies at lower REE concentrations. Further, this partitioning likely varies among the iron oxide solids that form^19,22,23,25^ which are controlled by an array of environmental parameters. In addition, adsorption processes also fractionate towards HREEs, although the effects are likely weak at environmental concentrations far from saturation of surface sites.^18^ It is unclear if similar incorporation-induced REE fractionations occur in marine sediments, where the iron oxide pool was recently shown to be enriched in LREEs *versus* HREEs.^72^ However, marine systems are confounded by early diagenetic effects that produce REE-phosphates,^73^ similar to the LREE-rich inclusions seen in some laterite and hydrothermal iron oxide systems.^10,11^ This also highlights the uncertain impacts of strongly-complexing ligands on REE substitution into iron oxides, conditions that may alter partitioning among dissolved, adsorbed, and structurally-bound pools.

## Conclusions

This study provides robust evidence for the incorporation of REEs into the crystal structure of goethite, demonstrating distinct mechanistic controls on REE partitioning dictated by ionic radius, coordination environments, and precursor states. HREEs such as Yb and Lu are preferentially incorporated into goethite, forming stable structural configurations associated with protonated Fe vacancies. These findings are consistent with prior reports of Lu substitution but extend the interpretation by providing multiple distinct configurations of Yb and Lu incorporation facilitated by substantial configurational disorder. LREEs and MREEs, specifically Nd and Dy, undergo limited structural incorporation into goethite, with substantial secondary amorphous REE phases detected.

At environmentally relevant conditions, Yb and Lu transition from ferrihydrite into goethite-like local environments even during partial transformation of ferrihydrite at ambient temperature, underscoring the intrinsic affinity of HREEs for the goethite structure. The goethite-like environment at low temperature is modeled well by the pure goethite samples, which indicates the configurations for Yb and Lu are similar for these two incorporation pathways. The slower kinetics of Lu incorporation compared to Yb, despite their similar ionic radii, reveal additional complexities related to electronic and magnetic structure effects. These observations suggest that HREE preference for incorporation into goethite extends across diverse formation pathways, including aging at room temperature and synthesis under alkaline and elevated temperature conditions.

The preferential retention of HREEs by goethite provides an explanation for observed environmental fractionations in regolith-hosted REE deposits, bauxites, and other weathering systems, where iron oxide phases are often enriched in HREEs. The identification of amorphous secondary phases associated with LREEs and MREEs offers new insights into exceptions to the typical HREE enrichment trends and suggests alternate pathways of REE retention in iron oxyhydroxide systems. These phases exhibit faster release during acid dissolution, potentially enabling targeted approaches for REE recovery and refining strategies tailored to separate LREE/MREE components from HREEs in iron oxide-hosted resources.

Moving forward, quantitative REE partitioning studies across the lanthanide series at lower concentration ranges are crucial for refining predictive models of fractionation effects during coprecipitation and aging of ferrihydrite. Additionally, partitioning relationships need to extend to other iron oxide phases formed under diverse environmental conditions. Adsorption processes, though weaker in fractionation effects, and competing complexation by strongly coordinating ligands also warrant further investigation to understand their impacts on REE incorporation and retention in iron oxyhydroxides. Finally, this work underscores the potential complexity of REE fractionation in sedimentary and hydrothermal systems, where diagenetic effects and competing phosphate formation may obscure trends observed in weathering environments. These insights provide a foundation for future investigations into REE behavior in iron oxides, with implications for resource recovery, environmental geochemistry, and the broader field of REE mobility and cycling in the critical zone.

## Conflicts of interest

There are no conflicts to declare.

## Supplementary Material

RA-015-D5RA04022A-s001

## Data Availability

The data supporting this article have been included as part of the SI. Table of synthesis conditions; SEM characterization; Rietveld refinement; Sequential dissolution; Estimated REE concentration profile; Goethite lattice parameters; EXAFS of NdGoe; X-ray scattering of ferrihydrite samples; Real-space fit of REEOOH to YbFH and LuFH EXAFS data; Shell-by-shell fit parameters for YbFH and LuFH; NdFH EXAFS data; X-ray scattering of aged FH samples; Aged NdFH EXAFS data; Comparison of NdGoe to literature data; Beam line configuration details; Magnetic state initialization approach. See DOI: https://doi.org/10.1039/d5ra04022a.
