# Supplementary material for: Structural incorporation into goethite fractionates rare earth elements
Source: RSC Adv. 2025 Aug 14;15(35):28815–26. doi: 10.1039/d5ra04022a (PMC12376894; doi:10.1039/d5ra04022a)
Supplement: RA-015-D5RA04022A-s001 [file RA-015-D5RA04022A-s001.pdf]

Supplementary Information (SI) for RSC Advances.
This journal is © The Royal Society of Chemistry 2025

Supplementary Information for:

Structural Incorporation into Goethite Fractionates Rare Earth Elements

Sebastian T. Mergelsberg,[1]* Alex J. Kugler,[1] Elaine D. Flynn,[2] Eric J. Bylaska,[1] Duo Song,[1] Jeffrey G. Catalano,[2]* Eugene S. Ilton[1]*

[1]Pacific Northwest National Laboratory, Richland, Washington 99354, United States

[2]Department of Earth and Planetary Sciences, Washington University, St. Louis, Missouri 63130, United States

*Author Contact: sebastian.mergelsberg@pnnl.gov
*Author Contact: catalano@wustl.edu
*Author contact: eugene.ilton@pnnl.gov

Table S1. Summary of sample labels used throughout the study. The use of room temperature (RT) refers to ambient laboratory temperature of 22 ± 2 °C.

Sample	Mineral phase	impurity	Synthesis pH	Synthesis temperature
RGoe	goethite	none	11	60 °C
NdGoe	goethite	Nd	11	60 °C
DyGoe	goethite	Dy	11	60 °C
YbGoe	goethite	Yb	11	60 °C
LuGoe	goethite	Lu	11	60 °C
RFH	ferrihydrite	none	6.8	RT
NdFH	ferrihydrite	Nd	6.8	RT
DyFH	ferrihydrite	Dy	6.8	RT
YbFH	ferrihydrite	Yb	6.8	RT
LuFH	ferrihydrite	Lu	6.8	RT

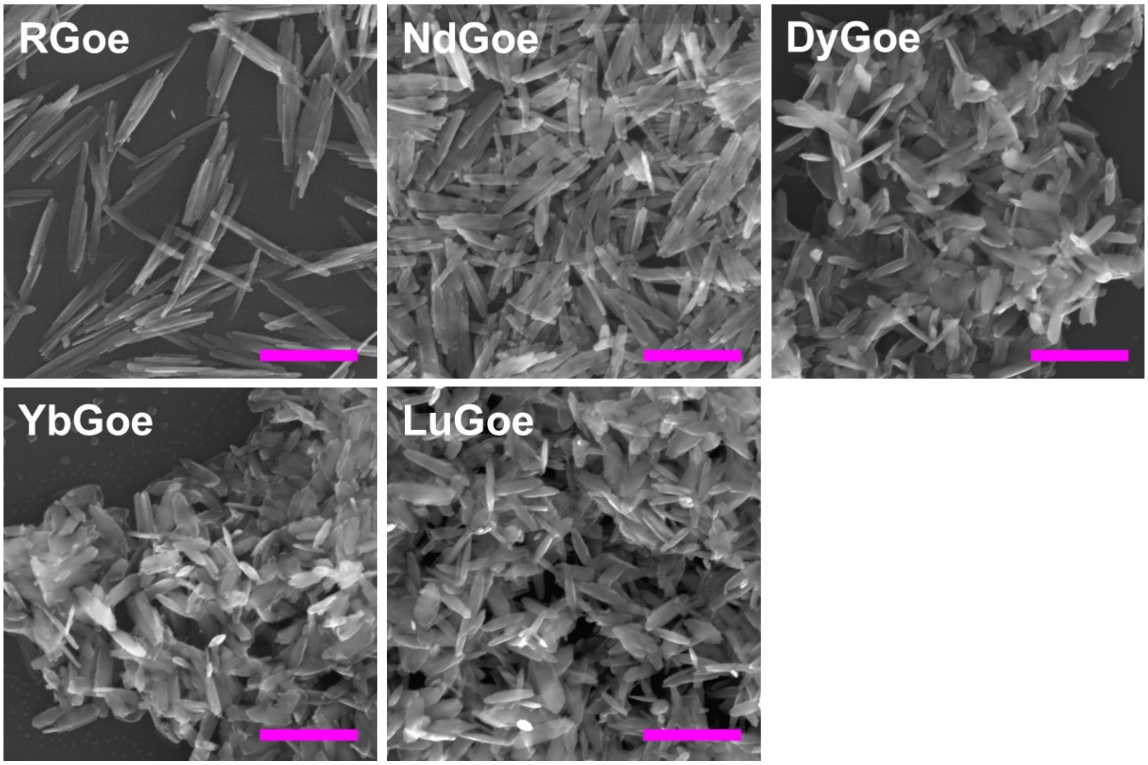

Figure S1. Scanning electron micrographs of the doped and undoped goethite samples. Addition of any REE visibly extends the particle width, which typically coincides with the *a*-direction (<100>), and shortens the longest axis, commonly assigned to the *b*-direction (<010>). The thickness of the goethite crystals is not resolved sufficiently to make any qualitative observations. All scale bars are 1 μm.

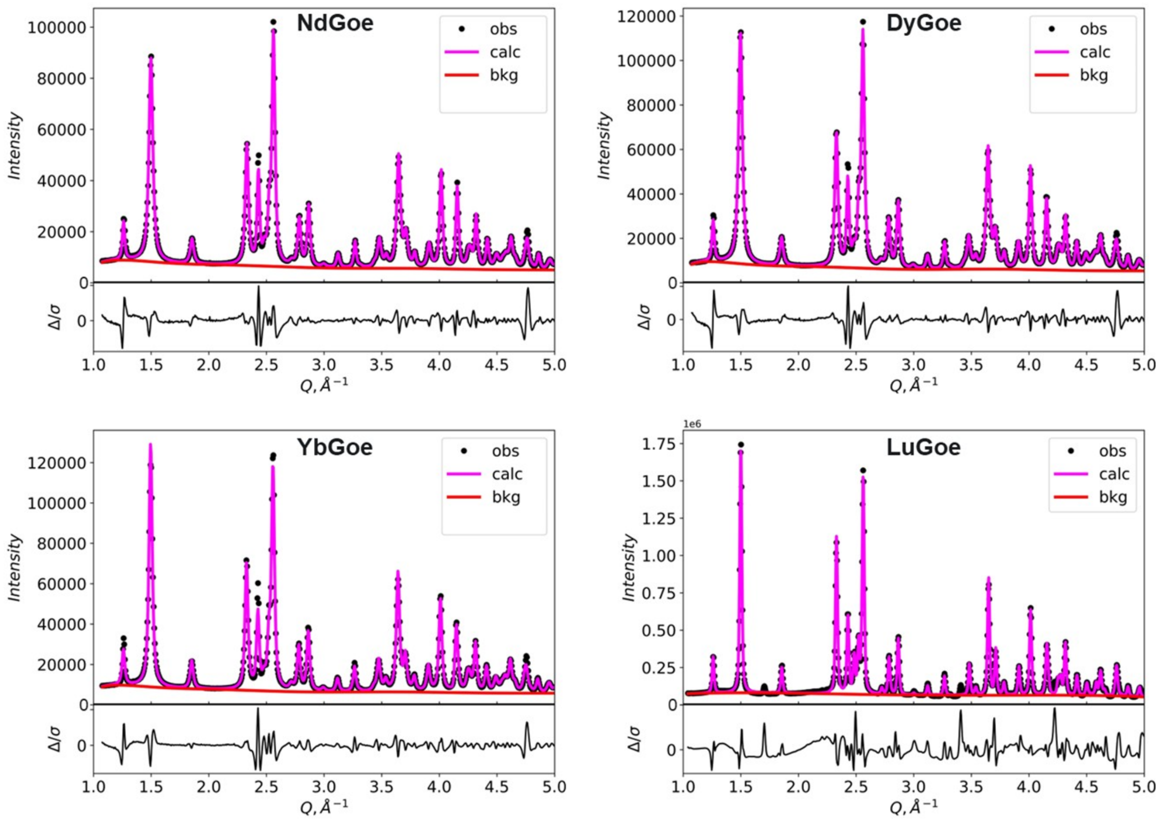

Figure S2. Rietveld refinement of heXRD data of all four doped goethite minerals. Note that the LuGoe was acquired during a separate beam time with a dilute ceria (CeO_2) standard measured in-line due to previous alignment issues (unfit peaks in residual, at 1.7, 3.45, and 4.2 $Å^{-1}$). In all four samples, the goethite reflection with the largest intensity error is the 210 at Q = 2.4 $Å^{-1}$, indicative of extra electron density of edge-sharing octahedra with two O(H) linkages.

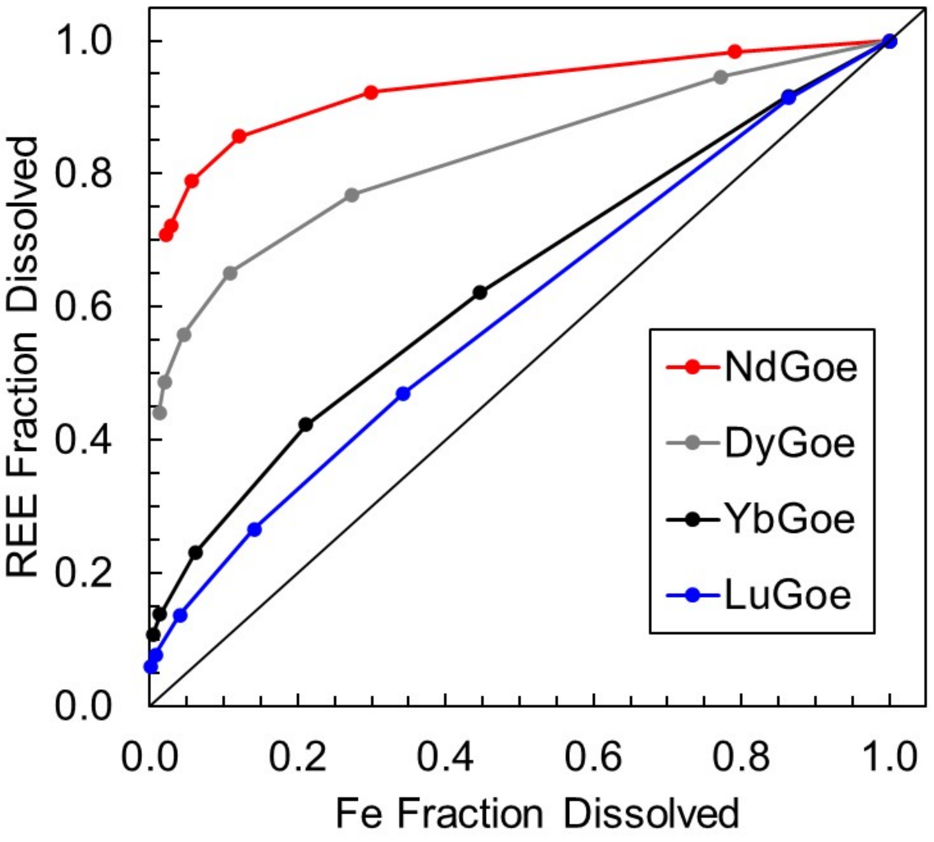

Figure S3. Sequential dissolution of all four goethite minerals. For each sample, the measured REE fraction is plotted against the measure Fe fraction. The grey y=x line is plotted for reference and represents the expected dissolution of a homogenously distributed impurity. Details of dissolution procedure in Materials and Methods.

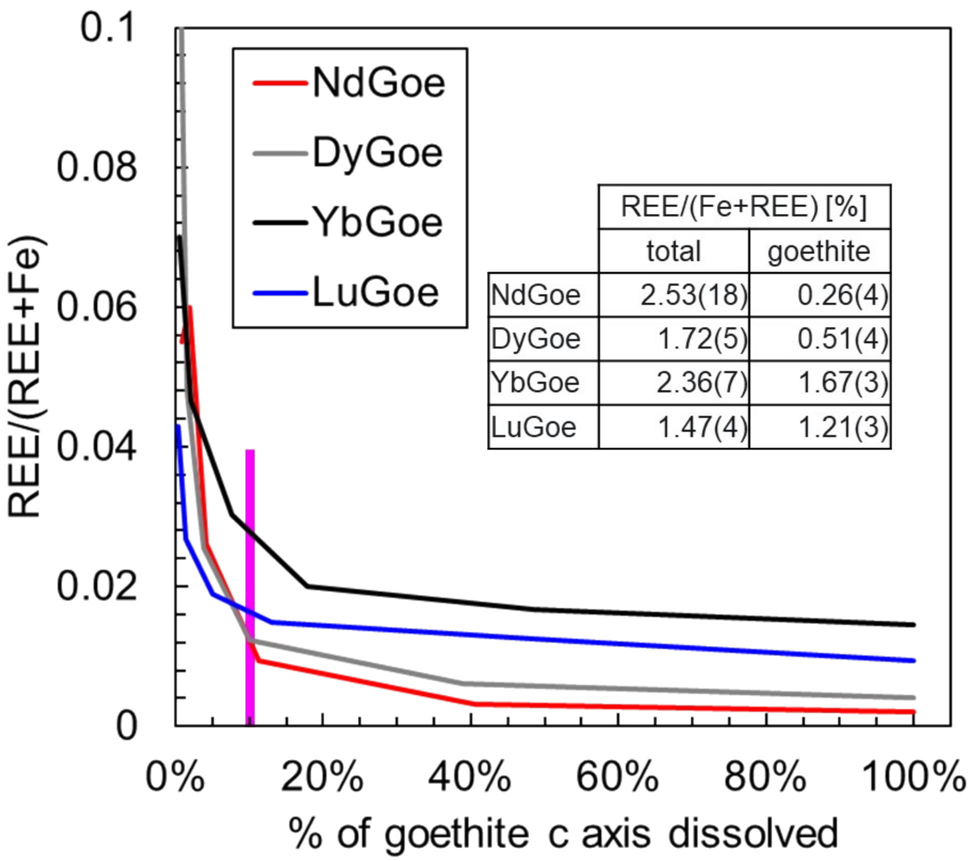

Figure S4. Numerical model of the fraction of REE over total Me vs. estimated % of c axis dissolved. This assumes an idealized goethite aspect ratio of 25:150:10 (a:b:c), where the short c-axis is perpendicular to the largest surface, and dissolution occurs congruent to the initial particle shape. The table gives total REE fractions and the average after 10% of the goethite c-axis is dissolved (pink line), where we reach a consistent composition (~0.5 nm off each of the two c-axis surfaces).

Table S2. Lattice parameters, unit cell volume V, and residual error estimate (R_{wp}) from the heXRD Rietveld refinement. The larger residual R_{wp} of LuGoe is a function of not fitting the in-line calibrant and the residual on the goethite scattering factor, R_F, is low and in-line with the other goethite minerals.

Mineral	*a* (Å)	*b* (Å)	*c* (Å)	V (Å^3)	R_{wp} (%)	R_F (%)
RGoe	9.9624(25)	3.0273(9)	4.6146(9)	139.171(48)	1.22	1.75
NdGoe	9.9541(5)	3.0242(1)	4.6201(3)	139.081(23)	3.38	3.67
DyGoe	9.9574(4)	3.0265(1)	4.6233(2)	139.327(8)	3.36	3.17
YbGoe	9.9646(4)	3.0296(1)	4.6277(1)	139.701(4)	5.43	5.74
LuGoe	9.9643(4)	3.0252(1)	4.6133(2)	139.545(6)	10.83	2.21

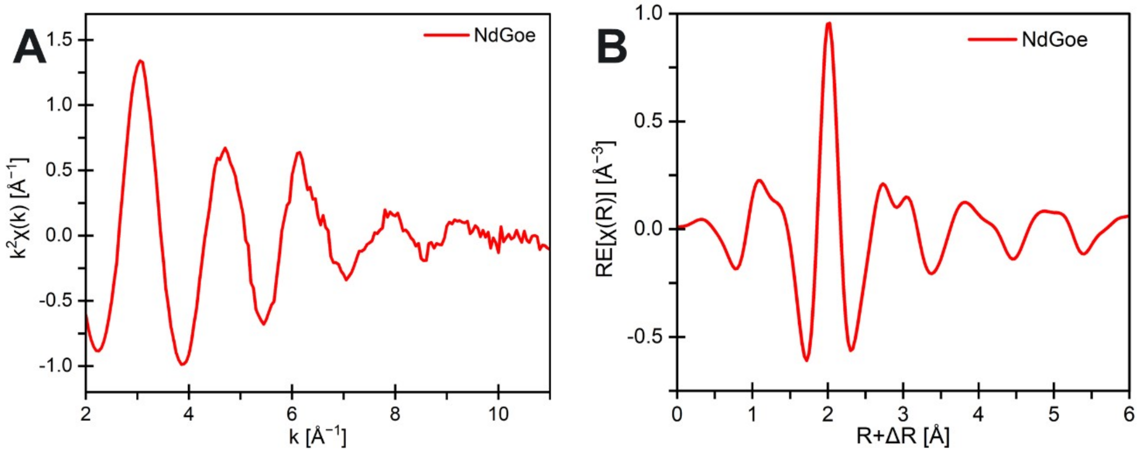

Figure S5. EXAFS of NdGoe. (A) Measurements of Nd L_{III}-edge in k-space and (B) FT-space.

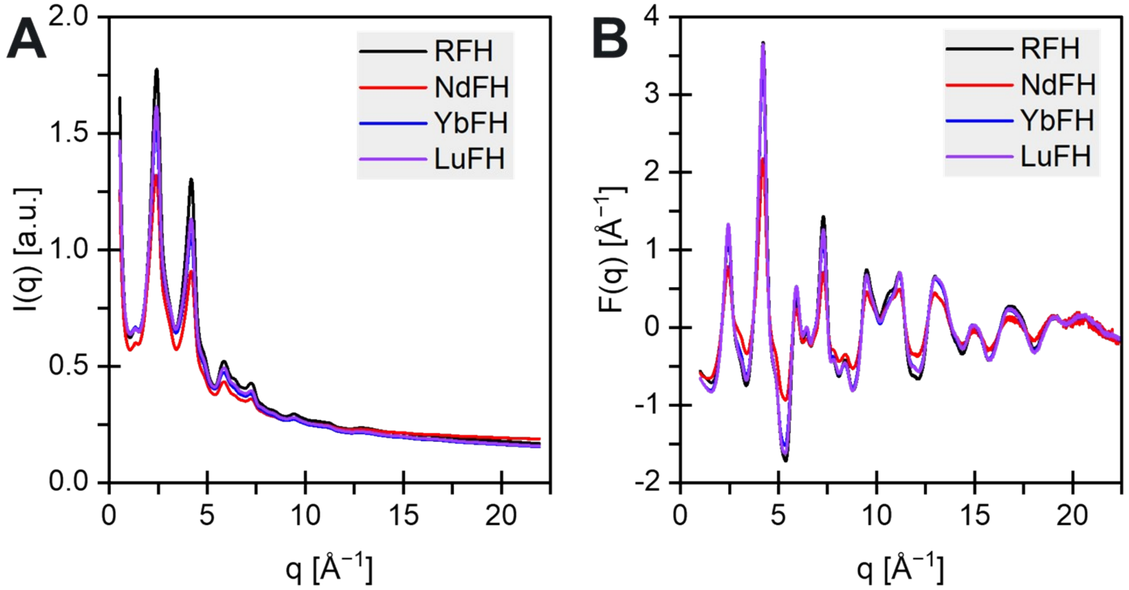

Figure S6. X-ray total scattering data of ferrihydrite samples, showing the measured intensities (A) and the normalized total structure factor F(q) (B).

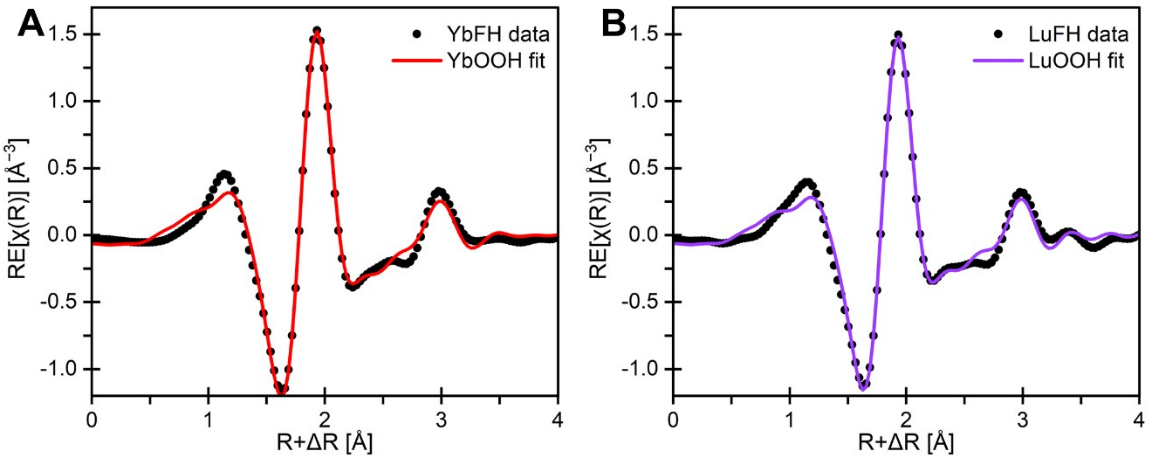

Figure S7. The real part of the Fourier transform from the shell-by-shell fit of (A) YbOOH to the YbFH EXAFS data and (B) the shell-by-shell fit of LuOOH to the LuFH EXAFS data. Fit results are reported in Table S2.

Table S3. Shell-by-shell fit parameters of REE-OOH structures for the YbFH and LuFH samples. Fits reported in Figure 6. Parameters in brackets were fixed.

Distance	YbFH	LuFH (Å)
Me─O(H) [CN = 7] (Å)	2.31 (2)	2.30 (1)
σ^2 (Å^2)	0.009(3)	0.009(2)
Me─Me_1 [CN = 6] (Å)	3.58 (7)	3.55 (3)
σ^2 (Å^2)	0.011(8)	0.012(4)
Me─Me_2 [CN = 4] (Å)	3.79 (11)	3.77 (5)
σ^2 (Å^2)	0.011(8)	0.012(4)

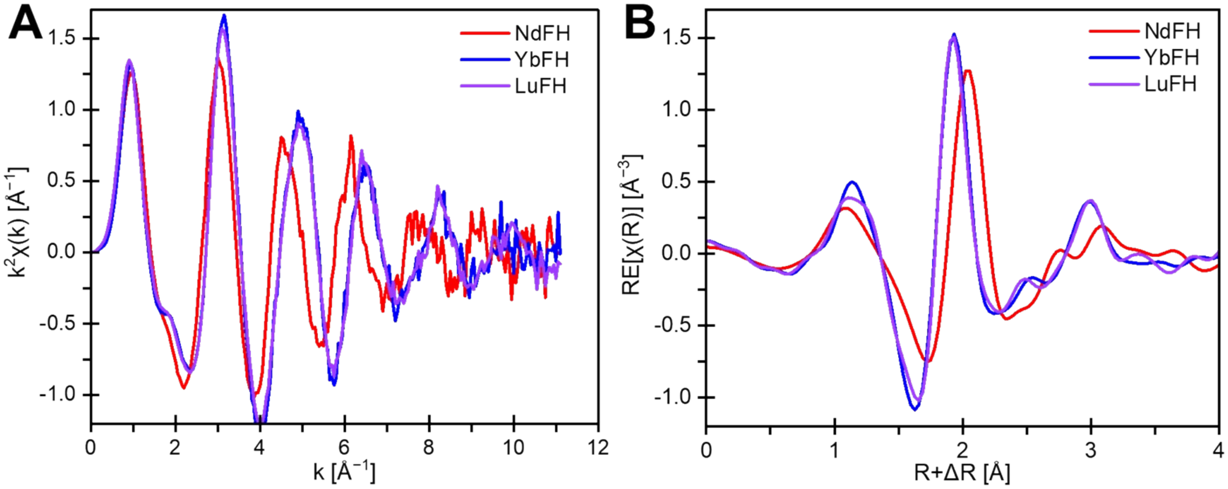

Figure S8. Comparison of FH data for Nd, Yb, and Lu. (A) K-space data and (B) the real part of the FT data, using 2.2 to 10.5 Å−1 as the FT window. Data show Nd forms a different phase from Yb and Lu with significantly larger bond lengths, indicative of a coordination number >7.

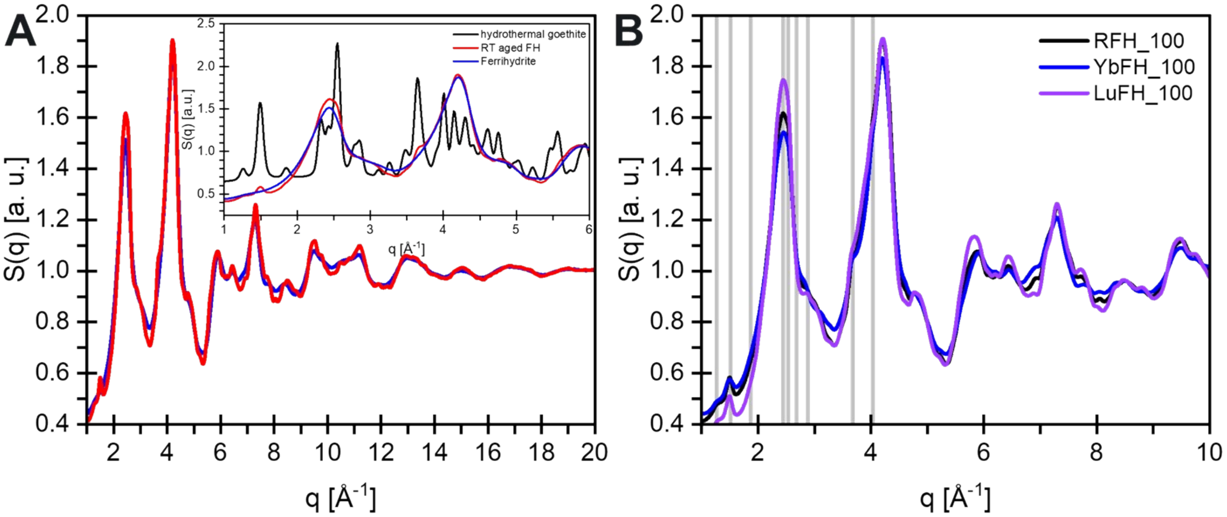

Figure S9. (A) Total structure factor [S(q)] of RT FH, hydrothermal goethite, and RT aged FH (100 days). The goethite peaks in the red pattern are indicative of ~5 vol% goethite. Quantification was not possible, due to the variable hydration state of ferrihydrite.(B) Comparison to total structure factors of doped ferrihydrite samples. The grey lines represent all goethite reflections with >10% relative intensity.[1]

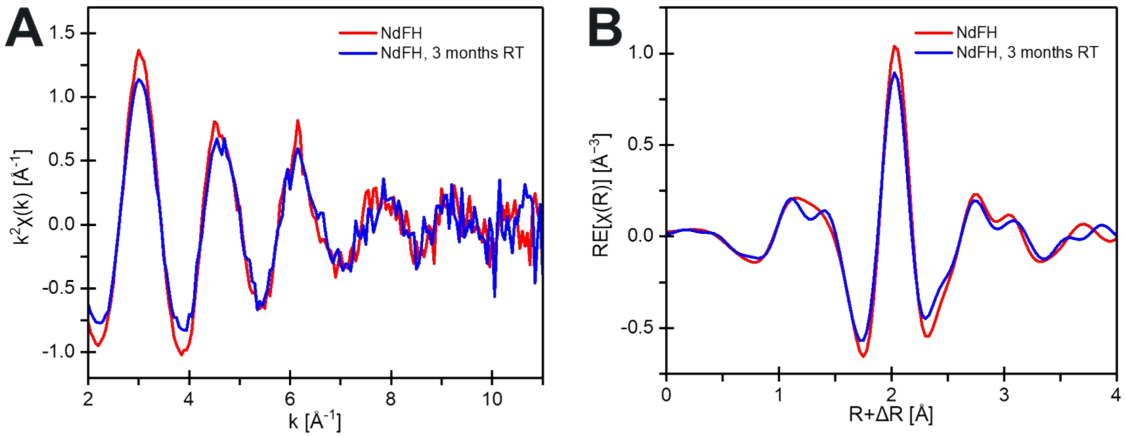

Figure S10. Comparison of Nd L_{III}-edge EXAFS of the FH samples before (red) and after (blue) 100 days ageing at RT. (A) Data comparison in k-space and (B) FT-space.

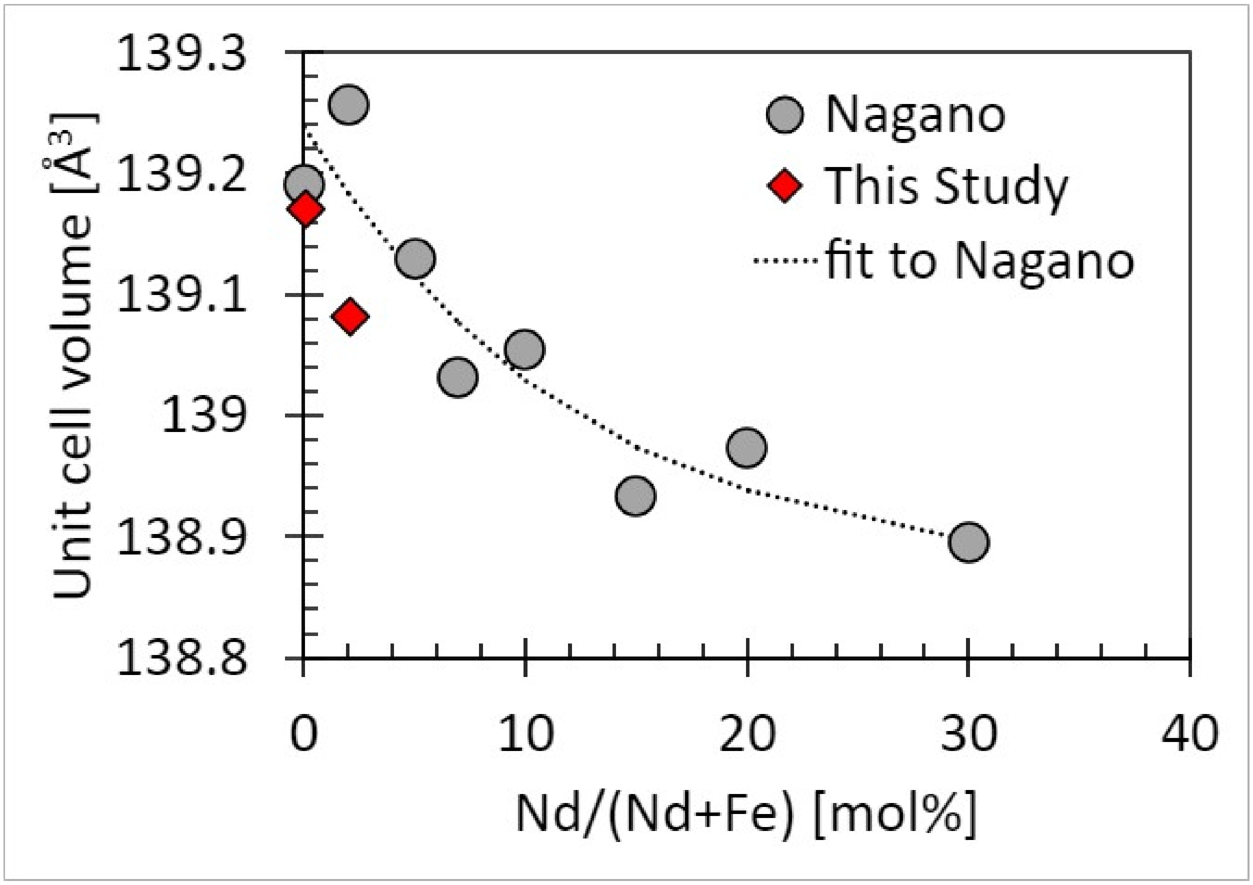

Figure S11. Comparison of refined unit cell volumes for NdGoe from this study (red diamonds) compared to the published data from Nagano et al.

Note S1. Beam line configurations for EXAFS measurements

Data collection at SSRL utilized beamlines 4-1, 4-3, and 11-2. Beamline 4-1 and 11-2 use Si (220) monochromators while beamline 4-3 employs a Si (111) monochromator. The monochromators were detuned 40% at the Nd L_3-edge and 30% at the Yb and Lu L_3-edge to decrease the harmonic content of the beam. A flat Si mirror was used at beamline 11-2 to further decrease harmonic content. Fluorescence-yield data were collected at SSRL using a 32-element energy-dispersive Ge detector (4-1), a 7-element Vortex silicon drift detector (4-3), or a 100-element segmented Ge detector (11-2). Measurements at NSLS-II utilized beamline 6-BM BMM, which is equipped with a Si (111) monochromator, a paraboloid collimating mirror before the monochromator, and a flat harmonic rejection mirror afterwards.

Note S2. Initializing the Magnetic State Using the Spin-Penalty Function Approach

Magnetic materials can exhibit a wide range of spin configurations, including ferromagnetic, antiferromagnetic, and more complex arrangements. Correctly initializing the desired magnetic state is essential for accurate Density Functional Theory (DFT) simulations, since it influences the convergence to a stable solution and the accuracy of the resulting electronic structure. Given the combinatorically large number of possible magnetic configurations, a systematic initialization approach is required. Here, we employ the spin-penalty function approach to reliably set up a specified magnetic configuration in DFT calculations using NWChem/NWChemEx.

The spin-penalty function approach is a method for stabilizing an initial magnetic configuration by selectively applying penalties on certain atoms to favor specific spin orientations. This method modifies the non-local part of the pseudopotential to impose spin-dependent potentials, encouraging the electronic wave functions to localize spins in the desired orientations on specific sites within the system. By stabilizing the target spin arrangement on neighboring atoms, the spin-penalty function approach can help guide symmetry of the initial wave function, promoting a convergence toward a desired magnetic ground state.

Spin-Penalty Function Approach

The spin-penalty function approach is implemented by transforming the pseudopotential energy term E_{psp} into a modified form $E_{psp-penalty}$, incorporating the spin penalties. The modified pseudopotential energy expression is written as:

$$E_{psp-penalty} = \sum_{\sigma=\uparrow,\downarrow}\sum_{i=1}^{n_{elc}^{\sigma}}\sum_{I=1}^{n_{ions}}\left(\langle\psi_i^{\sigma}|V_{local}^{I}|\psi_i^{\sigma}\rangle + \sum_{l=0}^{l_{max}^{I}}\sum_{m=-l}^{l}\sum_{n=1}^{n_{max}^{I}}\sum_{n'=1}^{n_{max}^{I}}\left(1-\delta_{l,l_I^{\sigma}}\left(\xi_{I,l}^{\sigma}-1\right)\right) \times \text{[illegible]}\right.$$

where $\xi_{I,l}^{\sigma}$ and l_I^{σ} specify the strength and locations of the penalties, respectively. The penalty term enforces spin localization on chosen atoms, creating an artificial stabilization of antiparallel spins on neighboring sites. This setup emulates the desired magnetic configuration by generating an initial state that favors the magnetic ordering across the unit cell, making it easier for the

system to converge to the magnetic ground state in subsequent DFT calculations. The detailed definition of each term in this formula is given in Table I.

Table 1: Terms in the Spin-Penalty Formula

**Symbol**	**Description**
$E_{psp-penalty}$	Total energy of the pseudopotential modified by the spin-penalty function.
σ	Spin index (σ = ↑ or ↓) representing spin-up or spin-down states.
n_{elc}^{σ}	Number of electrons with spin σ (either spin-up or spin-down).
i	Index of the electron in the sum.
I	Index of the ion (or atom) in the system.
V_{local}^{I}	Local part of the pseudopotential for atom I.
$\langle\psi_i^{\sigma}\vert V_{local}^{I}\vert\psi_i^{\sigma}\rangle$	Expectation value of the local pseudopotential for electron i with spin σ around atom I.
l	Orbital angular momentum quantum number, defining the orbital type.
l_{max}^{I}	Maximum angular momentum for which the pseudopotential includes projector functions for atom I.
m	Magnetic quantum number, defining the orientation of the orbital.
n,n'	Indices for different radial components of the projector functions within the same angular momentum channel.
n_{max}^{I}	Maximum number of radial components (or projector functions) for atom I in each l-channel.
$\delta_{l,l_I^{\sigma}}$	Kronecker delta function, used to apply the spin penalty selectively to specific angular momentum channels.
$\xi_{I,l}^{\sigma}$	Penalty strength for atom I in the angular momentum channel l with spin σ, enforcing spin alignment.
$P_{n'lm}^{I}$	Projector function for atom I with quantum numbers n, l, and m, part of the non-local pseudopotential.
$h_{l,n,n'}^{I}$	Matrix element that defines the interaction strength between different projector functions for atom I.
$\langle\psi_i^{\sigma}\vert P_{nlm}^{I}\rangle h_{l,n,n'}^{I}\langle P_{n'lm}^{I}\vert\psi_i^{\sigma}\rangle$	Overlap of the electron wave function ψ_i^{σ} with the projector functions, quantifying alignment with specific angular momentum states.

Steps for Initializing a Magnetic Configuration Using the Spin-Penalty Function Approach

The process for initializing a magnetic configuration (such as antiferromagnetic, ferromagnetic, or other spin-ordered states) using the spin-penalty function approach involves the following steps:

1. **Define the Magnetic Lattice and Identify Spin-Ordered Sites**:
   - Identify the atoms or ions in the lattice where specific spin orientations are desired. For instance, in antiferromagnetic configurations, neighboring atoms may

need opposite spins, whereas in ferromagnetic configurations, neighboring spins would align parallel.

- Select these atoms during setup, specifying which will receive spin penalties to achieve the intended magnetic order. Note that this selection process is not part of the formula itself but is a preparatory step.

2. **Specify the Multiplicity of the Electronic State**:
   - Set the spin multiplicity of the system based on the desired total spin state. The multiplicity (M=2S+1, where S is the total spin quantum number) controls the overall spin configuration of the electronic state, guiding the DFT calculation toward the correct magnetic state.
   - Specifying the multiplicity ensures that the total spin aligns with the target configuration, aiding convergence to the intended magnetic ground state.
3. **Assign Spin Penalty Values**:
   - For each chosen atom, assign a penalty strength $\xi_{I,l}^{\sigma}$ to enforce the desired spin orientation. This parameter influences the degree to which the penalty enforces spin localization.
   - Apply penalties selectively to different angular momentum components (e.g., d-orbitals for transition metals) by choosing the appropriate lll value, ensuring that the penalty function targets the magnetic orbitals relevant to the system's magnetic behavior.
4. **Run the Initial DFT Calculation with Spin Penalties Enabled**:
   - Execute a DFT calculation with the spin-penalty function activated. This calculation will generate an electronic structure with localized spins in the intended magnetic arrangement.
   - Monitor the spin densities to confirm that the chosen spin configuration has been successfully established on the designated atoms.
5. **Optimize the Structure with Penalties Disabled**:
   - Once the magnetic ordering is achieved, gradually reduce the spin penalty values and re-optimize the structure without the penalty terms.
   - This step allows the electronic structure to relax to a stable solution, where the spin ordering is now naturally maintained by the system without the artificial stabilization provided by the penalties.

Explanation of Multiplicity in the Spin-Penalty Context

Specifying the spin multiplicity aligns the overall spin state of the electronic structure with the desired magnetic configuration. In systems with multiple competing spin states, setting the multiplicity:

- It provides additional guidance to achieve the correct magnetic ground state.
- Helps ensure that the total spin in the system is consistent with the target configuration (e.g., singlet for antiferromagnetic or triplet for ferromagnetic arrangements).

- Complements the spin-penalty function approach by reducing the likelihood of convergence to an unintended spin state, especially in complex systems with competing magnetic configurations.

Advantages of the Spin-Penalty Function Approach for Magnetic Systems

The spin-penalty function approach offers several advantages for initializing magnetic configurations in with NWChem/NWChemEx plane-wave DFT calculations:

- **Systematic Control of Spin Configurations**: By applying penalties to specific atoms and orbitals, this approach allows for precise control over the initial spin structure. This flexibility makes it possible to explore a range of magnetic configurations—such as antiferromagnetic, ferromagnetic, or non-collinear states—and ensures reliable convergence to the intended magnetic state.
- **Improved Convergence:** The spin-penalty function approach helps direct the system toward a targeted magnetic state, reducing the likelihood that the system will settle in an unintended or metastable configuration. This is especially useful for systems with multiple competing spin states, achieving the correct magnetic configuration.
- **Enabling Studies of Complex Magnetic Materials**: The spin-penalty function approach is particularly valuable for materials with intricate magnetic interactions, such as frustrated spin systems, layered magnetic structures, and materials with competing magnetic ground states. This method provides a robust framework for initializing and stabilizing complex spin arrangements, facilitating accurate simulations of materials with unique and exotic magnetic properties.

References

1. A. F. Gualtieri and P. Venturelli, *Am. Mineral.*, 1999, **84**, 895-904.
